# A nomogram for predicting cancer-specific survival for elderly patients with gallbladder cancer

**DOI:** 10.1186/s12876-022-02544-y

**Published:** 2022-11-02

**Authors:** Chong Wen, Jie Tang, Tao Wang, Hao Luo

**Affiliations:** 1 General Surgery Center, The General Hospital of Western Theater, Chengdu, 610083 Sichuan Province China; 2 College of Medicine, Southwest Jiaotong University, Chengdu, China; 3 Department of Biostatistics and Epidemiology, School of Public Health, Shenyang Medical College, Shenyang, China

**Keywords:** Gallbladder cancer, Cancer-specific survival, Elderly patients, Nomogram, SEER

## Abstract

**Background:**

Gallbladder cancer (GBC) is a highly aggressive malignancy in elderly patients. Our goal is aimed to construct a novel nomogram to predict cancer-specific survival (CSS) in elderly GBC patients.

**Method:**

We extracted clinicopathological data of elderly GBC patients from the SEER database. We used univariate and multivariate Cox proportional hazard regression analysis to select the independent risk factors of elderly GBC patients. These risk factors were subsequently integrated to construct a predictive nomogram model. C-index, calibration curve, and area under the receiver operating curve (AUC) were used to validate the accuracy and discrimination of the predictive nomogram model. A decision analysis curve (DCA) was used to evaluate the clinical value of the nomogram.

**Result:**

A total of 4241 elderly GBC patients were enrolled. We randomly divided patients from 2004 to 2015 into training cohort (*n* = 2237) and validation cohort (*n* = 1000), and patients from 2016 to 2018 as external validation cohort (*n* = 1004). Univariate and multivariate Cox proportional hazard regression analysis found that age, tumor histological grade, TNM stage, surgical method, chemotherapy, and tumor size were independent risk factors for the prognosis of elderly GBC patients. All independent risk factors selected were integrated into the nomogram to predict cancer-specific survival at 1-, 3-, and 5- years. In the training cohort, internal validation cohort, and external validation cohort, the C-index of the nomogram was 0.763, 0.756, and 0.786, respectively. The calibration curves suggested that the predicted value of the nomogram is highly consistent with the actual observed value. AUC also showed the high authenticity of the prediction model. DCA manifested that the nomogram model had better prediction ability than the conventional TNM staging system.

**Conclusion:**

We constructed a predictive nomogram model to predict CSS in elderly GBC patients by integrating independent risk factors. With relatively high accuracy and reliability, the nomogram can help clinicians predict the prognosis of patients and make more rational clinical decisions.

**Supplementary Information:**

The online version contains supplementary material available at 10.1186/s12876-022-02544-y.

## Introduction

Gallbladder cancer (GBC) is a relatively rare but highly aggressive malignancy. Worldwide, GBC accounts for 1.2% of all cancer diagnoses and is the 22nd most common cancer, but 1.7% of all cancer deaths make it the 17th most deadly malignancy [1]. The incidence of GBC is higher in females than in males and in developing countries than in developed countries [2, 3]. Other risk factors for gallbladder cancer include obesity, family history, hepatitis virus infection, and most importantly, gallstones and chronic cholecystitis [2]. In recent years, the incidence of GBC is increasing in many countries and regions around the world, especially in Asia [4–7]. Furthermore, elderly GBC patients have a higher incidence, higher morbidity, and shorter overall survival [8, 9]. Gallbladder cancer patients are mainly middle-aged and elderly people. In the United States, about 97% of gallbladder cancer patients are 45 years and older, and 71% of gallbladder cancer patients are 65 years and older [10]. Therefore, the survival prognosis of elderly GBC patients deserves our careful attention.

As far as we know, the eighth American Joint Committee on Cancer (AJCC) staging system is more practical to predict all stages of gallbladder cancer and does not accurately estimate the prognosis of individual patients [11]. Thus, a nomogram that includes various factors is needed to predict the prognosis of a specific group of patients. In oncology and medical research, a nomogram is a common tool for assessing the prognosis of individual patients because of its friendly and feasible interface [12]. Up to now, many gallbladder cancer-related nomogram prediction models have been established [13–15]. However, to our knowledge, no nomograms have been developed specifically to predict a specific population of elderly patients with gallbladder cancer. Aiming at elderly GBC patients, it is necessary to establish a more targeted, reliable, and practical prediction model to predict CSS.

This study aimed to select prognosis-related risk factors and integrate them to construct a nomogram for predicting the cancer-specific survival of elderly GBC patients so that can help clinicians better judge prognosis and make clinical decisions.

## Materials and methods

### Data retrieved from SEER

**Fig. 1:**
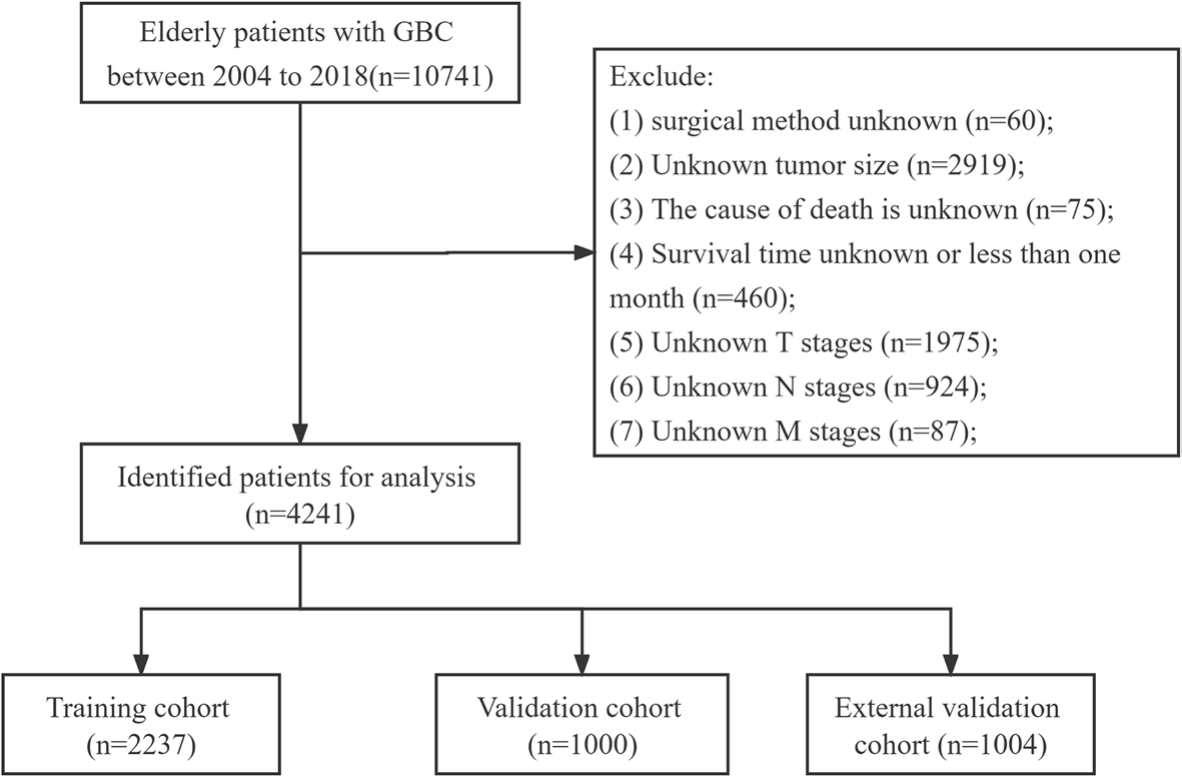
Flow chart of patient screening

Clinicopathological data and prognosis of all patients with GBC from 2004 to 2018 were retrieved from the SEER database. Considering that SEER is a public database from which anyone can access data, and private patient information is not identifiable, informed consent of patients or ethical approval is not required. We performed the analysis in accordance with the SEER database usage rules. Clinical variables that we collected included sex, age, race, the status of marriage, year of diagnosis, tumor stage, tumor profile, histological grade, tumor size, radiation, chemotherapy, and surgical method. The above clinical variables covered demographic characteristics, tumor information, and treatment modalities, which is very comprehensive. The inclusion criteria included: (1) The years of diagnosis were 2004–2018; (2) Aged 65 years or older; (3) Pathological diagnosis identified GBC. The exclusion criteria included: (1) Unknown tumor size; (2) Surgical method unknown; (3) Survival time unknown or less than 1 month; (4) The cause of death is unknown; (5) Unknown TNM stages. A total of 4241 elderly GBC patients were enrolled in this study. Patients with GBC diagnosed between 2004 and 2015 were randomly divided into the training cohort (70%) and the internal validation cohort (30%), and patients diagnosed between 2016 and 2018 were defined as the external validation cohort (Fig. 1).

### Nomogram development and statistical analysis

The specific statistical description and statistical analysis methods are consistent with our previous paper [16]. Briefly, the training cohort was used (*n* = 2237) to establish the nomogram and to predict the nomogram model using both the internal cohort (*n* = 1000) and external cohort (*n* = 1004). The independent risk factors of elderly GBC patients were selected by univariate and multivariate Cox proportional hazard regression analysis. Then a predictive nomogram model using all these risk factors was set up. Subsequently, the calibration curve, decision analysis curve (DCA), Consistency Index (C-index), and area under the receiver operating curve (AUC) were applied for validating the accuracy and discriminating the predictive nomogram model. Finally, based on the nomogram scores, the patients were divided into low-risk and high-risk groups and compared the differences in CSS between the two groups using survivorship curves. Statistical analysis was performed using R software 4.1.0 and SPSS 26.0. P< 0.05 was considered to be statistically significant.

## Result

### Clinical features

A total of 4241 patients with GBC diagnosed in 2004–2018 and aged 65 and older were included in the study. Among patients from 2004 to 2015, the average age was 76.6 ± 7.37 years, 2492 (77.0%) patients were white, 1014 (31.3%) patients were male, and 1541 (47.6%) patients were married. The histological grades I, II, III, and IV was 396 (12.2%), 1189 (36.7%), 1040 (32.1%), and 82 (2.53%), respectively. The remaining 530 (16.4%) have unknown stages. The histological type was adenocarcinoma in 2370 (73.2%) patients and non-adenocarcinoma in the rest of 867 (26.8%) patients. The distribution of histological subtypes of non-adenocarcinoma is shown in Fig. S1. There were 1290 patients (39.9%) diagnosed in the year 2004–2009 and the rest of 1947 patients (60.1%) in 2010–2015. There were 554 (17.1%), 1223 (37.8%), 1321 (40.8%), and 139 (4.29%) patients with stage T1, T2, T3, and T4 tumors, respectively. The mean tumor size is 38.0 ± 26.9 mm. Radical surgery was performed in 2132 (65.9%) patients, simple or partial surgery was performed in 545 (16.8%) patients, and 560 (17.3%) patients did not have any surgery. 1015 (31.4%) patients underwent chemotherapy and 2222 (68.6%) did not undergo chemotherapy or were unknown. 517 (16.0%) patients received radiation therapy, and 2720 (84.0%) received no radiation therapy or unknown. The clinical and pathological data of all patients are shown in Table 1.

**Table 1:** Clinicopathological characteristics of elderly patients with GBC

	All	Training cohort	validation cohort	
	***N*** = 3237	***N*** = 2237	***N*** = 1000	p
Age	76.6 (7.37)	76.5 (7.32)	76.9 (7.50)	0.170
Race				0.451
white	2492 (77.0%)	1709 (76.4%)	783 (78.3%)	
black	365 (11.3%)	256 (11.4%)	109 (10.9%)	
other	380 (11.7%)	272 (12.2%)	108 (10.8%)	
Sex				0.580
Male	1014 (31.3%)	708 (31.6%)	306 (30.6%)	
Female	2223 (68.7%)	1529 (68.4%)	694 (69.4%)	
Marital				0.101
Married	1541 (47.6%)	1087 (48.6%)	454 (45.4%)	
No	1696 (52.4%)	1150 (51.4%)	546 (54.6%)	
Year of diagnosis				0.697
2004–2009	1290 (39.9%)	897 (40.1%)	393 (39.3%)	
2010–2015	1947 (60.1%)	1340 (59.9%)	607 (60.7%)	
Histologic type				0.646
Adenocarcinoma	2370 (73.2%)	1632 (73.0%)	738 (73.8%)	
Non-adenocarcinoma	867 (26.8%)	605 (27.0%)	262 (26.2%)	
Grade				0.644
I	396 (12.2%)	282 (12.6%)	114 (11.4%)	
II	1189 (36.7%)	819 (36.6%)	370 (37.0%)	
III	1040 (32.1%)	704 (31.5%)	336 (33.6%)	
IV	82 (2.53%)	57 (2.55%)	25 (2.50%)	
Unknown	530 (16.4%)	375 (16.8%)	155 (15.5%)	
T				0.098
T1	554 (17.1%)	388 (17.3%)	166 (16.6%)	
T2	1223 (37.8%)	847 (37.9%)	376 (37.6%)	
T3	1321 (40.8%)	894 (40.0%)	427 (42.7%)	
T4	139 (4.29%)	108 (4.83%)	31 (3.10%)	
N				0.261
N0	2292 (70.8%)	1570 (70.2%)	722 (72.2%)	
N1	945 (29.2%)	667 (29.8%)	278 (27.8%)	
M				0.742
M0	2479 (76.6%)	1709 (76.4%)	770 (77.0%)	
M1	758 (23.4%)	528 (23.6%)	230 (23.0%)	
Tumor size	38.0 (26.9)	37.8 (26.9)	38.3 (26.9)	0.635
Surgery				0.034
No	560 (17.3%)	402 (18.0%)	158 (15.8%)	
Simple/partial surgery	545 (16.8%)	394 (17.6%)	151 (15.1%)	
Radical surgery	2132 (65.9%)	1441 (64.4%)	691 (69.1%)	
Chemotherapy				0.217
No/Unknown	2222 (68.6%)	1520 (67.9%)	702 (70.2%)	
Yes	1015 (31.4%)	717 (32.1%)	298 (29.8%)	
Radiation				0.454
No/Unknown	2720 (84.0%)	1872 (83.7%)	848 (84.8%)	
Yes	517 (16.0%)	365 (16.3%)	152 (15.2%)	

### Independent risk factors in the training cohort

We used univariate and multivariate Cox proportional hazard regression models in the training cohort to identify the risk factors of elderly GBC patients. Univariate Cox regression analysis found that age, TNM stage, tumor size, histological grade of the tumor, surgical methods, and chemotherapy were associated with prognosis. While sex, marital status, race, year of diagnosis, and histological type were not statistically significantly associated with prognosis. All the risk factors screened by the univariate Cox analysis were included in the multivariate Cox regression analysis, and the results showed that all the included factors were independent risk factors for patient prognosis (Table 2).

**Table 2:** Univariate and multivariate analyses of GBC in training cohort

	Univariate			Multivariate		
	HR	95%CI	P	HR	95%CI	P
Age	1.01	1–1.02	0.005	1.021	1.014–1.029	< 0.001
Race						
white	reference					
black	1.07	0.91–1.26	0.436			
other	0.91	0.78–1.07	0.267			
Sex						
Male	reference					
Female	0.96	0.86–1.07	0.451			
Marriage						
No	reference					
Married	1.06	0.95–1.17	0.287			
Year of diagnosis						
2004–2009	reference					
2010–2015	0.94	0.85–0.15	0.274			
Grade						
I	reference			reference		
II	1.63	1.33–1.99	< 0.001	1.367	1.113–1.68	0.003
III	2.75	2.25–3.37	< 0.001	1.805	1.465–2.224	< 0.001
IV	3.3	2.32–4.69	< 0.001	2.272	1.585–3.256	< 0.001
Unknown	4.23	3.4–5.25	< 0.001	1.481	1.153–1.902	0.002
T						
T1	reference			reference		
T2	1.18	0.99–1.42	0.068	1.266	1.051–1.526	0.013
T3	3.83	3.23–4.55	< 0.001	2.789	2.323–3.348	< 0.001
T4	6.08	4.69–7.88	< 0.001	3.024	2.294–3.987	< 0.001
N						
N0	reference			reference		
N1	1.86	1.67–2.07	< 0.001	1.362	1.208–1.535	< 0.001
M						
M0	reference			reference		
M1	3.78	3.37–4.24	< 0.001	2.061	1.797–2.365	< 0.001
Tumor size	1.01	1.01–1.01	< 0.001	1.003	1.001–1.005	0.012
Surgery						
No	reference			reference		
Simple/partial surgery	0.23	0.19–0.27	< 0.001	0.5	0.403–0.62	< 0.001
Radical surgery	0.24	0.21–0.27	< 0.001	0.49	0.409–0.586	< 0.001
Chemotherapy						
No/Unknown	reference			reference		
Yes	0.83	0.72–0.95	0.008	0.702	0.619–0.795	< 0.001
Radiation:						
No/Unknown	reference					
Yes	1.3	1.17–1.45	< 0.001			

### Construction and validation of the prognostic nomogram

**Fig. 2:**
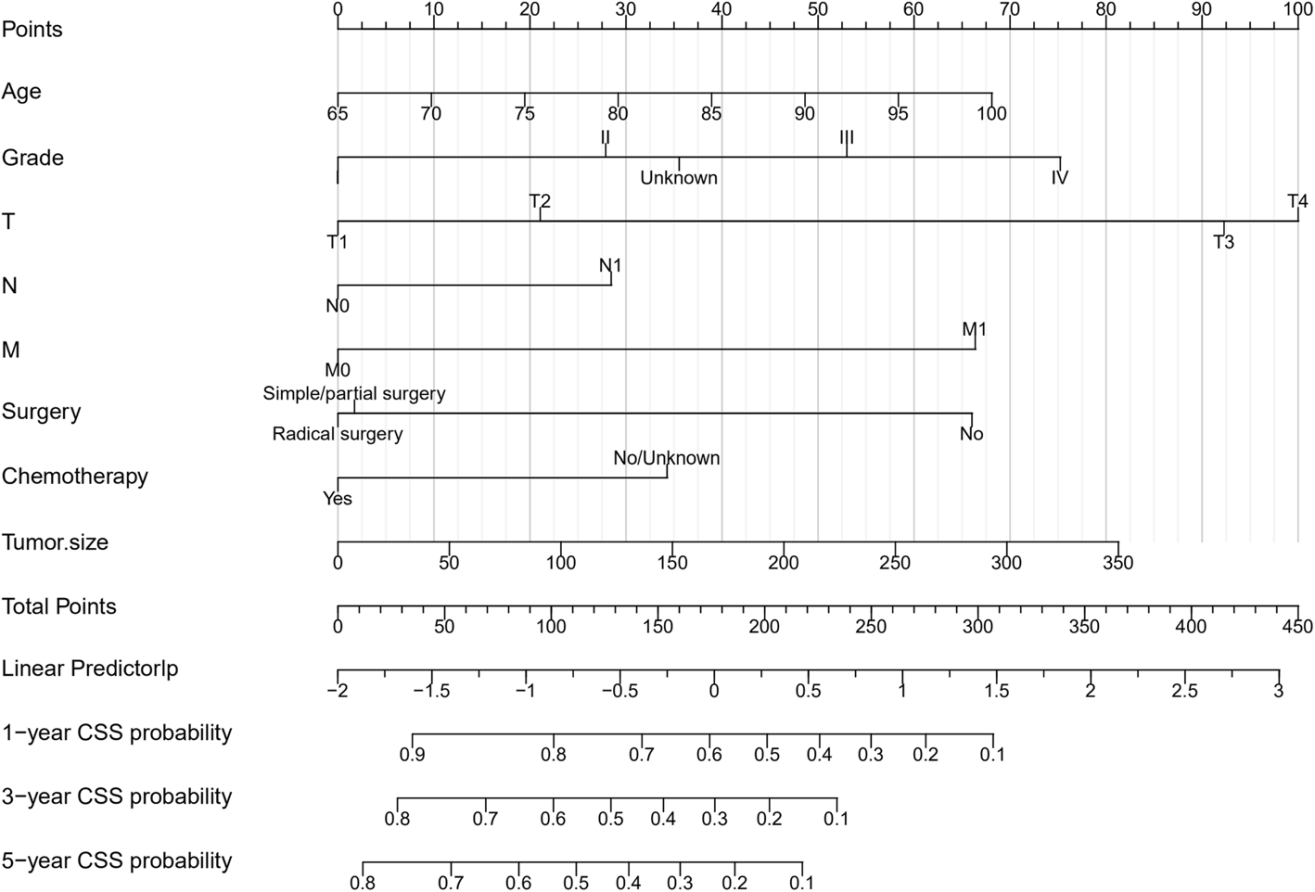
The nomogram for predicting 1-, 3-, and 5-year CSS in middle-aged GBC patients

**Fig. 3:**
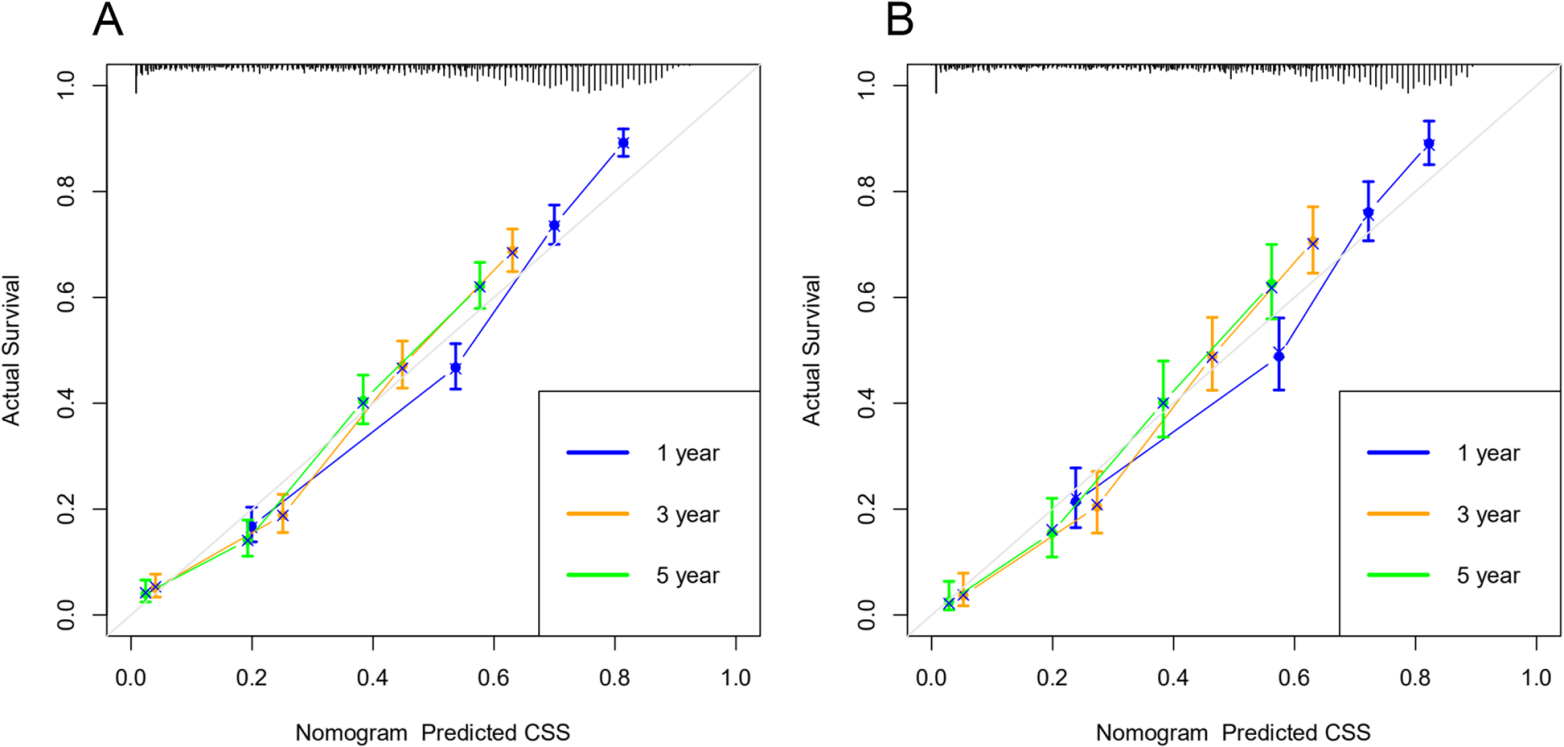
Calibration curves for comparing survival predicted by the model with actual observed survival. **A** training cohort calibration curves for 1 -, 3 - and 5-year CSS; **B** internal validation cohort calibration curves for 1-, 3-, and 5-year CSS

**Fig. 4:**
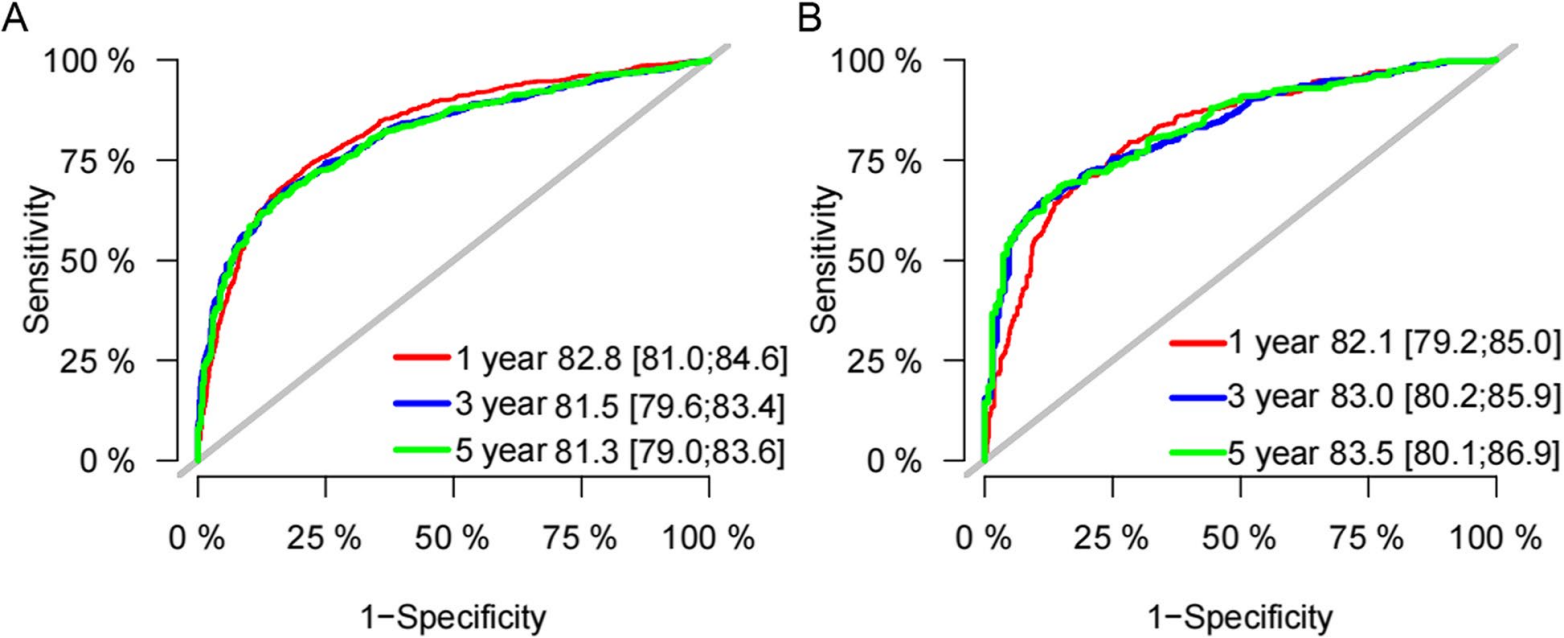
Receiver operating characteristic curve for predicting 1-, 3-, and 5-year CSS of elderly GBC patients. **A** receiver operating characteristic curve in the training cohort; **B** receiver operating characteristic curve in the internal validation cohort

We included all the independent risk factors to build a nomogram for predicting 1-, 3-, and 5-year CSS in elderly GBC patients (Fig. 2). As the nomogram shows, T stage and tumor size were the most statistically significant risk factors affecting cancer-specific survival, after that tumor histological grade, age, distant metastasis, and surgery. Besides, chemotherapy and lymph node metastasis also affect patient outcomes. The C-index of the nomogram was 0.763[0.757–0.769], 0.756[0.746–0.766], and 0.786[0.774–0.798] in the training, validation, and external validation cohort, respectively. It manifested that the prediction model had a good prediction ability. In both the training cohort and the internal validation cohort, the calibration curves show that the predicted values of the nomogram agree well with the observed values (Fig. 3), which confirmed its high accuracy. Besides, the receiver operating characteristic curve also indicated the high authenticity of the prediction model (Fig. 4).

### Clinical application of the nomogram

**Fig. 5:**
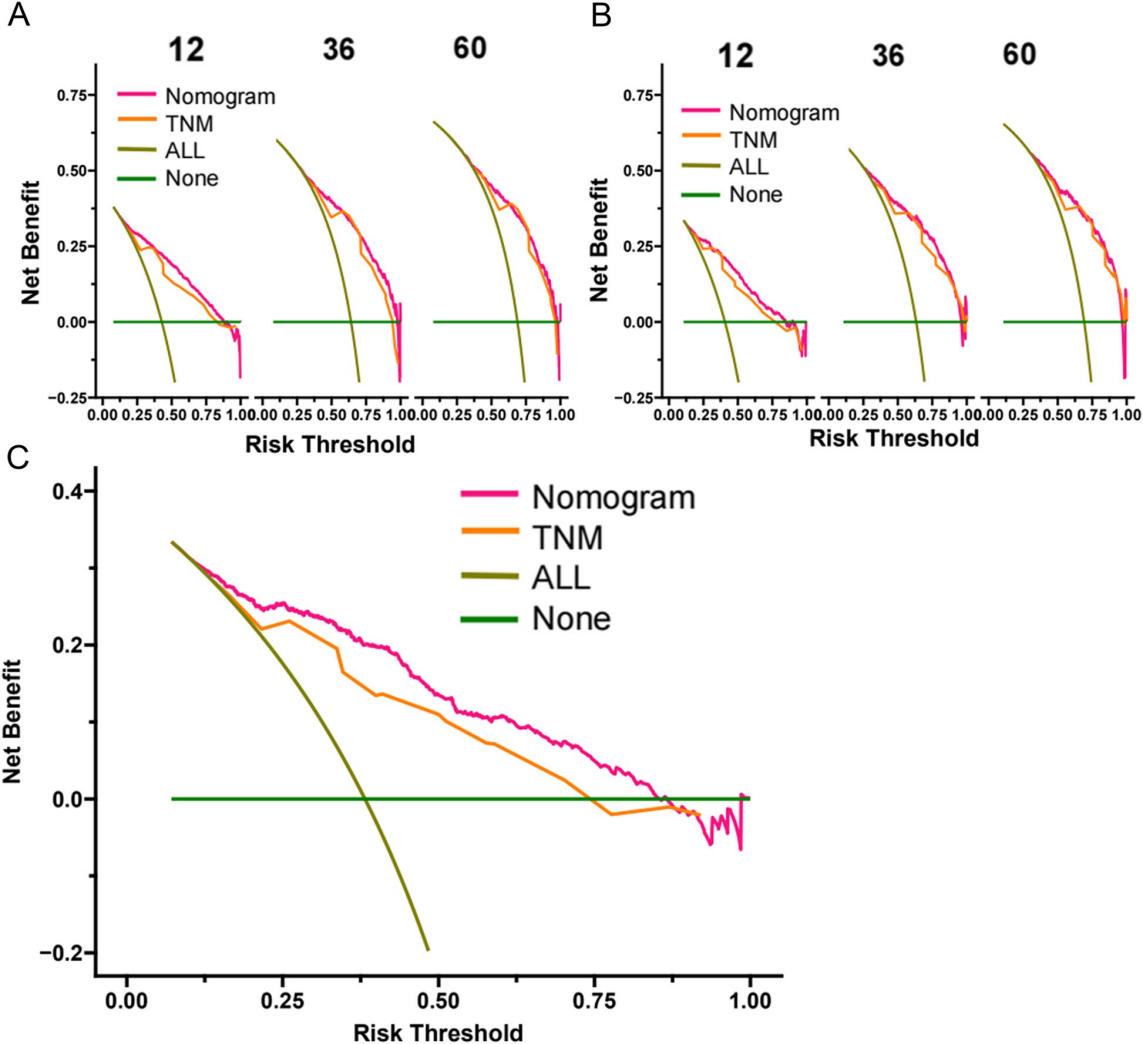
Decision curve analysis of the nomogram in the training cohort (**A**), validation cohort (**B**), and external validation cohort (**C**). The Y-axis represents a net benefit, and the X-axis represents threshold probability. The green line means no patients died, and the dark green line means all patients died. When the threshold probability is between 25 and 80%, the net benefit of the model exceeds all deaths or none

**Fig. 6:**
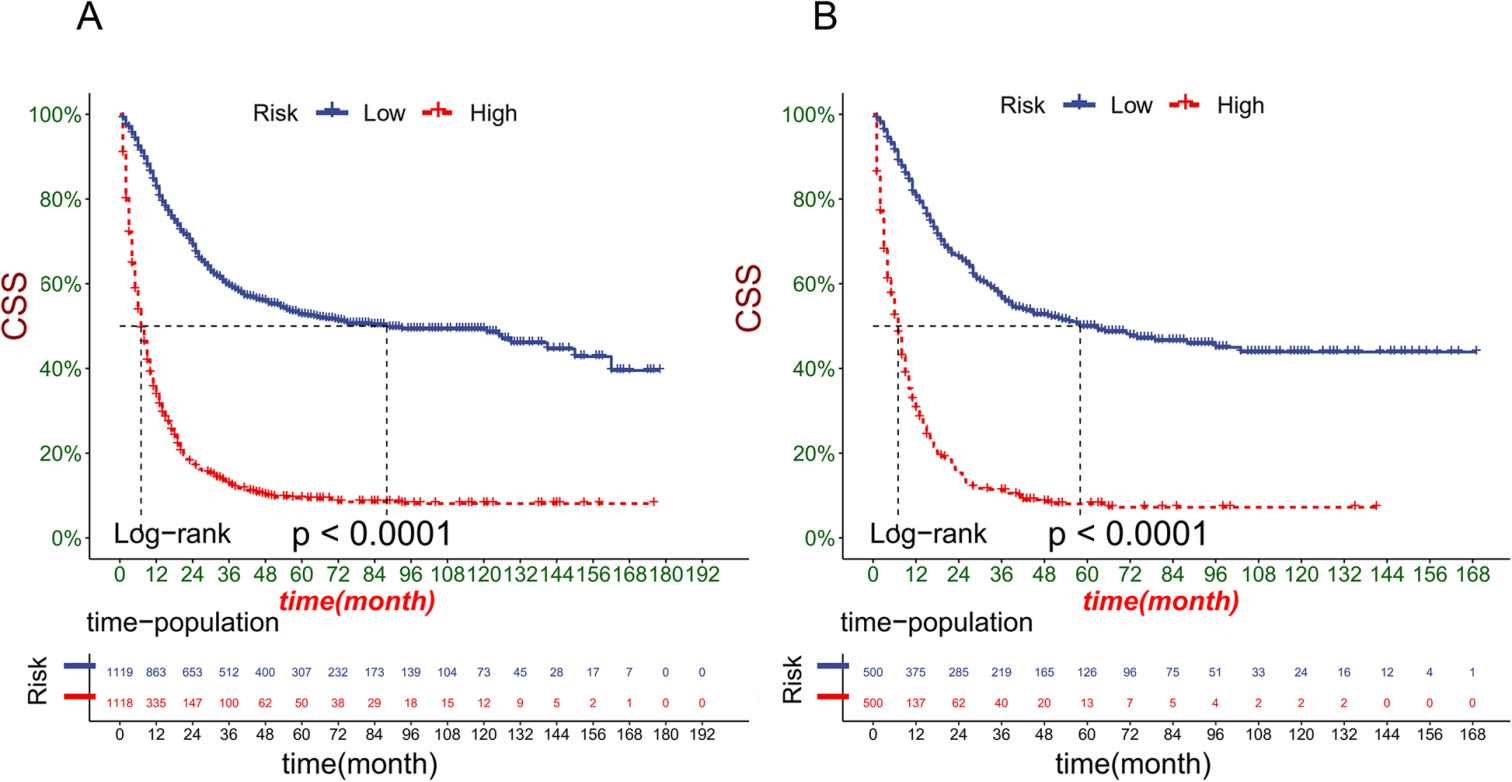
Kaplan-Meier curves for patients in the training cohort (**A**) and validation cohort (**B**)

**Fig. 7:**
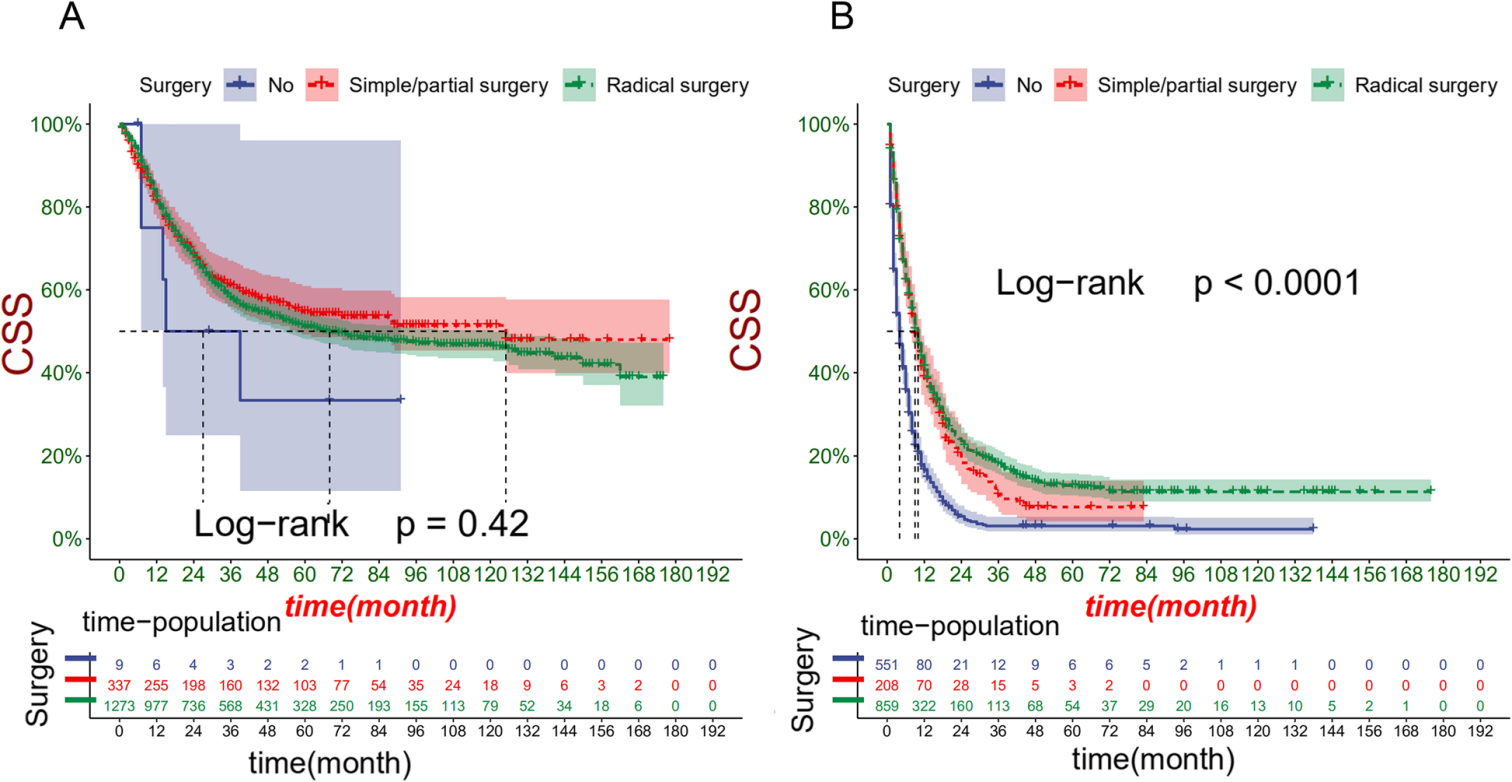
Kaplan-Meier curves for patients in the low-risk group (**A**) and high-risk group (**B**) of which undergone different surgical procedures

DCA manifested that the nomogram model had better prediction ability than the conventional TNM staging system (Fig. 5). All patients were divided into the high-risk group (total > 168) and the low-risk group (total ≤ 168) according to the individual score of the nomogram. In the training and validation cohort, survival was significantly higher in the low-risk group than in the high-risk group (Fig. 6). The 1-year, 3-year and 5-year survival rates in the high-risk group were 32.7, 12.2, and 9.0%, respectively. The 1-year, 3-year and 5-year survival rates in the low-risk group were 82.1, 58.7, and 51.8%, respectively. In the low-risk group, the effect of the surgical method on survival was not statistically significant (Fig. 7A). In the high-risk group, the survival of patients who received radical surgery was the highest (Fig. 7B).

## Discussion

Nomogram as a prediction tool is commonly used to estimate prognosis in oncology and medicine [12]. By far, it has been used in various cancers, such as liver [17–19], pancreas [20, 21], kidney [22, 23], and bone [24–26] et al. Besides, Bai et al. [13] constructed a nomogram to predict overall survival after gallbladder cancer resection in China. Cai et al. constructed a nomogram to predict distant metastasis in T1 and T2 gallbladder cancer [14]. Chen et al. developed a nomogram to predict overall survival in node-negative gallbladder cancer patients [15]. Nevertheless, the survival prognosis of patients with gallbladder cancer varies greatly with age, and elderly patients with GBC have higher morbidity and shorter overall survival. Therefore, we developed and validated the nomogram based on patients, tumor characteristics, and treatment methods to estimate the CSS of elderly GBC patients.

In our study, we made statistical descriptions and statistical analyses of elderly GBC patients from the SEER database. We randomly divided patients diagnosed between 2004 and 2015 into the training cohort and the internal validation cohort, and patients diagnosed between 2016 and 2018 were defined as the external validation cohort. There was no statistical difference between the training group and the validation group regarding age, race, year of diagnosis, histological grade, TNM stage, chemotherapy, and so on. Univariate and multivariate Cox analysis found that numerous factors significantly affected CSS, including age, histological tumor grade, TNM stage, surgery, chemotherapy, and tumor size. While other variables such as sex, marriage, year of diagnosis, race, and histological type were not identified as prognostic significance. Our study shows that the difference between the elderly group of GBC patients and whole GBC patients does exist, and further investigation is required.

There have been some developments in the treatment concept for patients with GBC. Previous studies show that surgery is the only treatment modality associated with a benefit in terms of survival [27, 28]. However, recent studies found that other treatment methods such as chemotherapy, targeted therapy, and immunotherapy can also produce benefits for gallbladder cancer patients [29], this is partly because of the development of sequencing technology and new drugs so that the treatment can be more precise. In addition, Mao et al. also found that “Surgery + Chemotherapy” treatment can provide survival benefits for patients with advanced GBC [30]. Recent studies have reported that the widely-used gemcitabine in combination with oxaliplatin (GEMOX) or cisplatin and tegafur in combination with oxaliplatin (SOX) is an effective chemotherapy regimen for patients with gallbladder cancer [30–32]. Our study found that patients who undergo chemotherapy have a better prognosis, paralleling recent studies. Nevertheless, surgery remains the cornerstone treatment for GBC patients. In our study, simple/partial surgery and radical surgery have similar benefits. In fact, there are only 25%–30% of total GBC patients undergo radical resection, partly due to many patients with gallbladder cancer are admitted to the hospital at an advanced stage and approximately two-thirds of gallbladder cancers are diagnosed accidentally in patients undergoing surgery for gallstones and cholecystitis (also known as “incidental gallbladder cancer, or IGBC”), inevitably leading to partial resection and simple surgery in patients, respectively [30, 33].

Among the nomogram tumor characteristics (pathological grade, TNM stage, tumor size), the T stage has the most significant effect on CSS. The risk of the T3 and T4 stages is significantly higher than that of the T1 and T2 stages. Lim et al. found that in patients with GBC after surgical resection, the TNM stage was the most important prognostic factor [34]. Groot et al. found that the prognosis of GBC patients depends strongly on the T stage, as well as the presence of lymph node metastasis, surgical resection margin, and tumor differentiation [35]. Compared with previous studies, our results consistently reflect the relationship between the T stage and CSS and quantify its impact on CSS in specific populations. Notably, age remains an important prognostic factor for patients aged 65 years or older with gallbladder cancer. This may be attributed to GBC being mostly diagnosed in the elderly, with the average age of GBC diagnosis in the US being 72 [2], which further proved the necessity of our research.

Our nomogram showed that in the training cohort, the AUC of 1-, 3-, 5-year CSS is 0.828 (95%CI:0.810–0.846), 0.815 (95%CI:0.796–0.834), and 0.813 (95%CI:0.790–0.836) respectively. In the internal validation cohort, the AUC of 1-, 3-, 5-year CSS is 0.821 (95%CI:0.792–0.850), 0.830 (95%CI:0.802–0.859), and 0.835 (95%CI:0.801–0.869) respectively. It indicates that our nomogram predicts the CSS of elderly GBC patients with a high degree of authenticity. In addition, DCA curves were also drawn to evaluate the clinical application value of the nomogram which showed that compared with the traditional TNM staging method, the nomogram could more accurately predict the CSS of elderly GBC patients at 1-, 3- and 5 years.

Nevertheless, there are some limitations in our study that should be considered. First, our study was based only on the SEER database. The results are not necessarily fully representative of other parts of the world outside of the USA, such as Asia, Africa, South America, etc. Second, the predictive nomogram does not include important factors such as tumor marker CA199, molecular factors, and the results of genetic diagnosis. Third, the data of our external validation cohort is also from the SEER database, which inevitably leads to relatively weak validation.

## Conclusion

Through statistical description and analysis, we found that the independent risk factors for tumor-specific survival in elderly patients with gallbladder cancer included age, tumor histological grade, TNM stage, surgical method, chemotherapy, and tumor size. More importantly, we constructed a predictive nomogram model to predict CSS in elderly GBC patients by integrating these risk factors. With relatively high accuracy and reliability, the nomogram can help clinicians predict the prognosis of patients and make more rational clinical decisions.

## Supplementary Information

**Additional file 1: Figure S1.** Histological type of gallbladder carcinoma in all patients. The vast majority of patients were adenocarcinoma, followed by squamous cell carcinoma, endocrine carcinoma, and some unknown epithelial tumors.

## Data Availability

The SEER data analyzed in this study is available at https://seer.Cancer.gov/.
