# Supplementary figures and images for: A nomogram for predicting cancer-specific survival for elderly patients with gallbladder cancer
Source: BMC Gastroenterol. 2022 Nov 2;22:444. doi: 10.1186/s12876-022-02544-y (PMC9632126; doi:10.1186/s12876-022-02544-y)

## Histological type

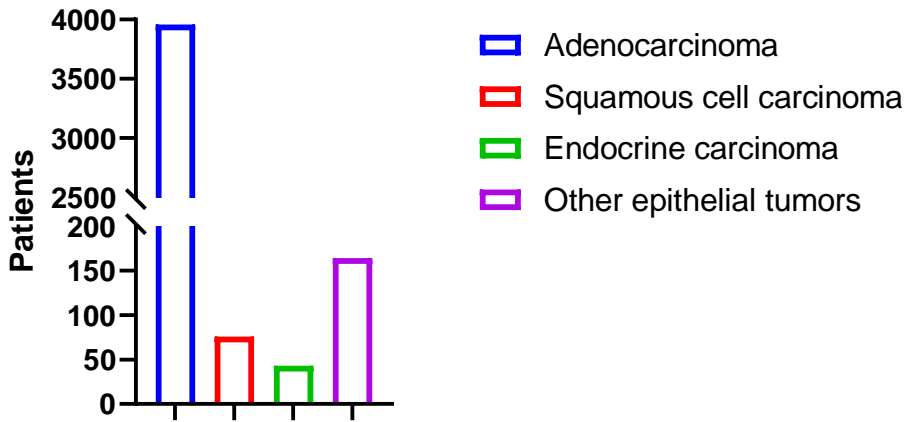

Supplement: Supplementary file 1 — Additional file 1: Figure S1. Histological type of gallbladder carcinoma in all patients. The vast majority of patients were adenocarcinoma, followed by squamous cell carcinoma, endocrine carcinoma, and some unknown epithelial tumors. [file 12876_2022_2544_MOESM1_ESM.pdf]
